# Non-dispersive sensing scheme based on mid-infrared LED and differential mode excitation photoacoustic spectroscopy

**DOI:** 10.1016/j.pacs.2023.100455

**Published:** 2023-01-20

**Authors:** J.M. Rey, M.W. Sigrist

**Affiliations:** ETH Zurich, Institute for Quantum Electronics, Laser Spectroscopy and Sensing Laboratory, Otto-Stern-Weg 1, Zurich CH-8093, Switzerland

## Abstract

A robust and simple sensing scheme utilizing a Mid-Infrared Light Emitting Diode (MIR-LED) and based on Differential Mode Excitation Photoacoustic (DME-PA) spectroscopy is presented. A MIR-LED light source in combination with optical correlation is used for simplicity and compactness. The sensing setup takes advantage of the non-linearity in the excitation of various acoustic modes in a cylindrical resonant photoacoustic cell to provide a high selectivity. The sensing device is tested using methane and hydrocarbon mixtures (propane, butane). The obtained limit of detection for methane is 25 ppm m^−1^. Using the presented DME-PA scheme, the derived gas concentration is hardly affected neither by intensity fluctuations of the light source nor by any microphone or electronics drifts. Furthermore, a considerably improved selectivity is obtained compared to conventional Non-Dispersive Infrared (NDIR) techniques.

## 1 Introduction

Thanks to their high sensitivity, large dynamic range, and comparatively simple experimental set-up, photoacoustic (PA) gas sensors are well-established sensing devices [1], [2], [3], [4], [5], [6]. The PA effect consists in the conversion of absorbed light energy into acoustical waves, which can be detected with microphones. PA sensors measure directly the small fraction of light absorbed by the gas sample within the PA cell. A great variety of PA cell designs have been proposed and applied to trace gas monitoring: acoustically resonant PA cells [7], [8], [9], eventually equipped with a resonance locking circuitry [10] or combined with a special resonance frequency tracking method [11]; open cells [12], [13]; cells with special geometry [14], [15]; multipass cells [16]; heatable cells [17]; intracavity cells [18]; parallel cells [19] and multifunctional, compact and miniaturized cells [20], [21], [22]. 3-D printing has also been applied to the fabrication of miniature PA cells [23]. Noise has been minimized by the development of ‘‘windowless’’ cells [24] and differential cells [25], [26]. Alternatively, all-optical PA spectrometers have been introduced [27], [28]. Further innovative developments concern quartz-enhanced photoacoustic spectroscopy, named QEPAS, pioneered by Kosterev et al. [29] and recently reviewed by Patimisco et al. [30]. After its introduction, QEPAS has subsequently seen numerous modifications and optimizations [31], [32], [33], [34], [35], [36], [37], as well as combinations with other configurations [38], [39]. A general problem of QEPAS, namely the dependence of energy relaxation processes in a gas mixture of their composition has recently been addressed in detail [40]. Finally, cantilever-enhanced photoacoustic spectroscopy, often named CEPAS has been introduced [41], [42], and successfully applied to gas sensing [43], [44], [45].

In most known PA sensing schemes, the absolute values of the microphone signal are measured and used to derive the concentration of the gas of interest [4, 5 and references therein]. Since PA signals depend on the intensity of the excitation light source used, some kind of intensity normalisation schemes (power meter or reference cells) are required for quantitative measurements. To reduce the complexity and the bulkiness associated with the use of normalization schemes, PA gas sensors based on Differential Mode Excitation Photoacoustic (DME-PA) spectroscopy have been proposed [46], [47], [48], [49]. The DME-PA method is based on the excitation of two different modes in a resonant PA cell and the gas concentration is derived from the amplitude ratio of these acoustic modes. The DME-PA technique also reduces the impact of microphone and electronics drifts on the measured gas concentration. This scheme has been utilized with black-body emitters [46], [48] and for the development of a gas sensor for water vapour using near-infrared light emitting diodes [47], [49].

Optical correlation spectroscopy provides a means of selectively detecting gases using broadband light sources. This technique uses a cell filled with the gas of interest as a selective spectral filter or detector. Although not as fundamentally sensitive as laser-based detection systems that offer superior spectral purity and higher power density, it has many real-world advantages. It covers a large wavelength range, is simple and cost-effective and, unlike laser systems, it is relatively immune to the problems of coherent sources that can exhibit multi-path interference effects (fringes). Optical correlation combined with PA spectroscopy enabled the development of selective gas detectors [50], [51], [52].

This paper presents a robust and simple sensing scheme based on DME-PA utilizing a Mid-Infrared Light Emitting Diode (MIR-LED) and optical correlation. The MIR-LED source is used for its simplicity, low power consumption and compactness. DME-PA in combination with optical correlation provides the selectivity, stability and the robustness. Measurements using methane and hydrocarbon mixtures (propane, butane) are presented to demonstrate the potential of the proposed sensing scheme.

## 2 Experimental arrangement

**Fig. 1:**
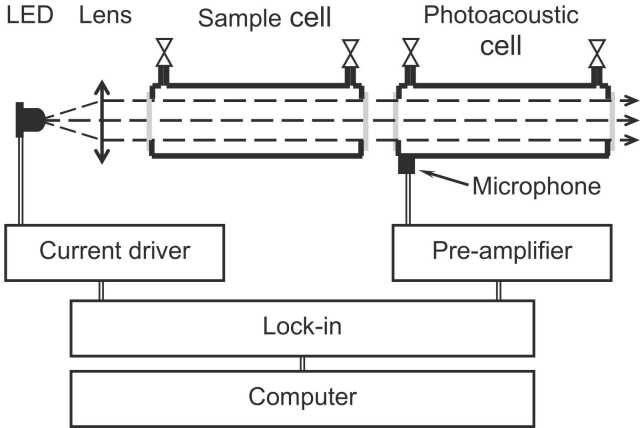
Schematic representation of the PA arrangement used in this work. The PA cell consists of a cylinder with length of 39 mm and diameter of 10 mm filled with 5.6%vv of CH4 in N2 at atmospheric pressure (980 mbar). The sample cell with the same diam. of 10 mm is 50 mm long and contains, among others, the gas species whose concentrations are to be determined.

**Fig. 2:**
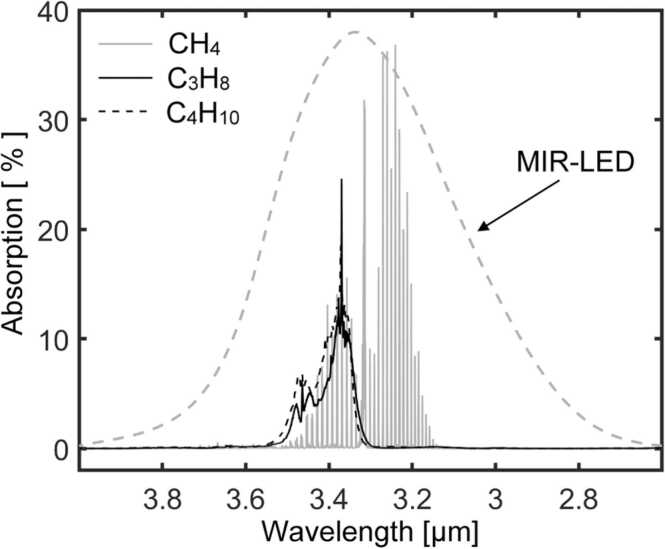
Absorption spectra taken from the PNNL database [53] of methane (CH4), propane (C3H8) and butane (C4H10) (each 1%vv, pathlength 1 cm, atmospheric pressure) as well as emission spectrum in arbitrary units (broken gray line) of the MIR-LED.

The experimental arrangement is depicted in Fig. 1. The excitation light source consists of a MIR-LED (LED34Sr, Ioffe Light Emitting Diode) equipped with a silicon immersion lens and having a peak emission wavelength at 3.35 µm and an emission FWHM of 0.5 µm well adapted to the methane absorption spectrum (Fig. 2). The MIR-LED temperature is set to 18 °C with a homemade temperature controller using a thermoelectric element. The LED drive current is sine-modulated between 0 and 200 mA corresponding to 0–200 μW of emitted power (measured with a powermeter (Ophir Laserstar 3A-SH)). The emitted light is collected and collimated with a CaF_2_ Lens (focal length 25 mm, diam. 10 mm). The infrared beam passes through a 50 mm long sample cell containing the sample gas whose concentration is to be determined before reaching the PA cell. The PA cell consists of a cylinder with a length of 39 mm and a diameter of 10 mm, filled with 5.6%vv of CH_4_ in N_2_ at atmospheric pressure (980 mbar). After passing the lens and the windows (all made of uncoated CaF_2_), the average light power entering the PA cell is about 40 μW and ca. 2.5 μW is absorbed by the methane present in the PA cell. The central cylinder is equipped with a miniature (diam. 2.57 mm) electret microphone (Knowles FG-23329, sensitivity: 22.4 mV/Pa at 10 kHz) located close to the entrance window of the PA cell (Fig. 1). The microphone signal is pre-amplified and monitored by a lock-in amplifier (Stanford Research Systems SR830). The MIR-LED current amplitude and frequency are controlled by a computer while the PA signal amplitude is transferred to the computer and recorded. To test and assess the performance of the sensing device, various gas mixtures were generated and filled into the sample cell by diluting a certified reference gas mixture of 40%vv methane in N_2_ (purity 5.0) as well as by diluting a butane (86%vv) / propane (14%vv) mixture in N_2_. The dilution process is conducted with a mass flow controller (Sierra, Serie800).

## 3 Results and discussion

**Fig. 3:**
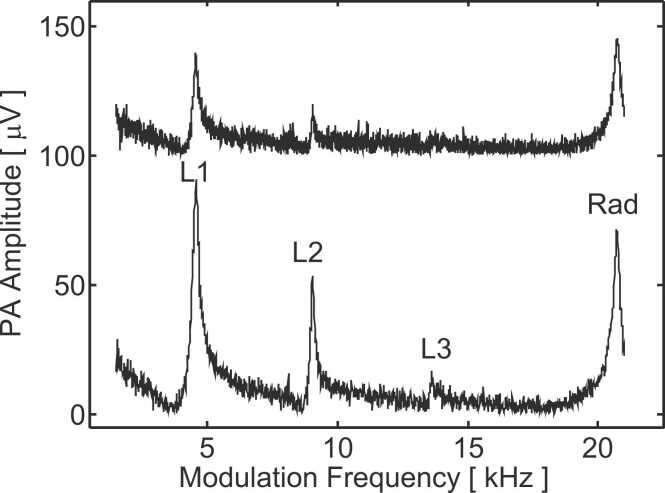
Frequency response of the PA cell filled with 5.6%vv of CH4 diluted in N2 at atmospheric pressure. The lower line corresponds to the sample cell filled with pure N2. The upper line is vertically shifted for better clarity and is obtained with the sample cell filled with 2%vv of CH4 diluted in N2 at atmospheric pressure. "L1", "L2" and "L3" indicate the positions of the first three longitudinal modes and "Rad" that of the first radial mode.

**Fig. 4:**
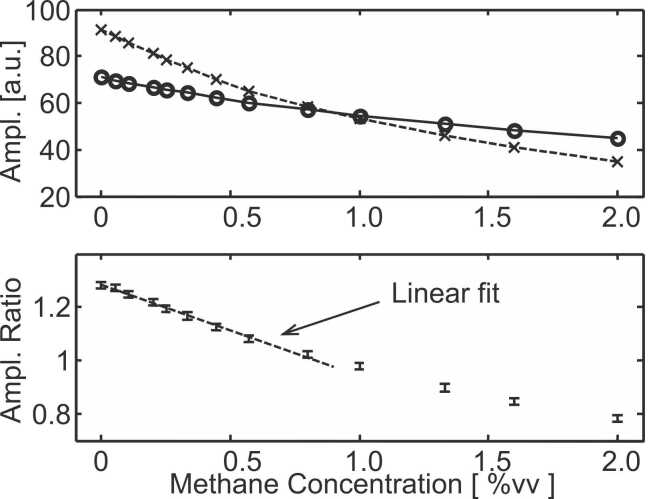
Amplitudes (upper part) and the corresponding ratio (lower part) of the first longitudinal “L1″ (x symbols) and radial “Rad” (o symbols) modes for various methane concentrations in the sample cell. The error bars in the lower part correspond to the experimental error (+/- 1 σ) of the ratios and the dashed line presents a linear fit within the linear range.

The frequency response of the PA cell filled with 5.6%vv of methane is shown in Fig. 3. The lower line presents the PA amplitude for various modulation frequencies with the sample cell filled with pure N_2_. The upper line is artificially shifted in the vertical direction for better clarity and corresponds to the amplitude measured with the sample cell filled with 2%vv of CH_4_ diluted in N_2_. When no absorption occurs in the sample cell (lower line in Fig. 3), the obtained PA signal is stronger and four resonances are observed. The three first ones (L1 at 4.5 kHz, L2 at 9.0 kHz and L3 at 13.6 kHz) correspond to longitudinal resonance modes and harmonics thereof while the fourth one (Rad at 20.7 kHz) corresponds to the first radial mode of the cylindrical PA cell. Fig. 3 implies that the relative intensity of the modes differs for the lower (sample cell filled with pure N_2_) and the upper (sample cell filled with 2%vv of methane) curve. As an example, the first longitudinal mode (L1) is stronger than the radial mode (Rad) for the lower curve while the opposite occurs for the upper one. This shows that the ratio of the mode amplitude (L1/Rad) depends on the methane concentration in the sample cell. Fig. 4 presents the maximum amplitude of the radial and of the first longitudinal resonances for various methane concentrations in the sample cell. Both the longitudinal and radial amplitudes decrease with increasing methane content in the sample cell but this decrease is much less pronounced for the radial mode (Rad) than for the longitudinal one (L1). This results in a mode amplitude ratio (i.e. the amplitude of the first longitudinal (L1) divided by the amplitude of the radial mode (Rad)) that depends on the methane concentration in the sample cell (Fig. 4). The methane concentration can thus be determined by measuring the mode amplitude ratio. The experimental noise attached to the PA signal amplitude is ca. 1% (1σ, 5 s integration time) and the experimental noise attached to the mode amplitude ratio is also 1% (1σ, 10 s integration time since two amplitudes have to be determined). Increasing the integration/averaging time to 40 s reduces the experimental noise on the ratio to ca. 0.5%. The dashed line in Fig. 4 is a linear fit (least square regression) of the experimental data points at low methane concentration where a linear behaviour is observed. Based on the slope of this linear fit of − 0.034 [ ^0^/_00_vv ^−1^], also called sensitivity, and the noise (1%) attached to the ratio determination, a limit of detection of 500 ppm (1σ, 20 s integration time) is obtained. Since the length of the sample cell is 50 mm, this corresponds to a limit of detection of 25 ppm m^−1^ for methane. Since the proposed device utilizes a LED light source which cannot be collimated along more than ca. 20 cm, the 25 ppm m^−1^ limit of detection should be considered as a way to compare the sensor performance with other sensing schemes.

**Fig. 5:**
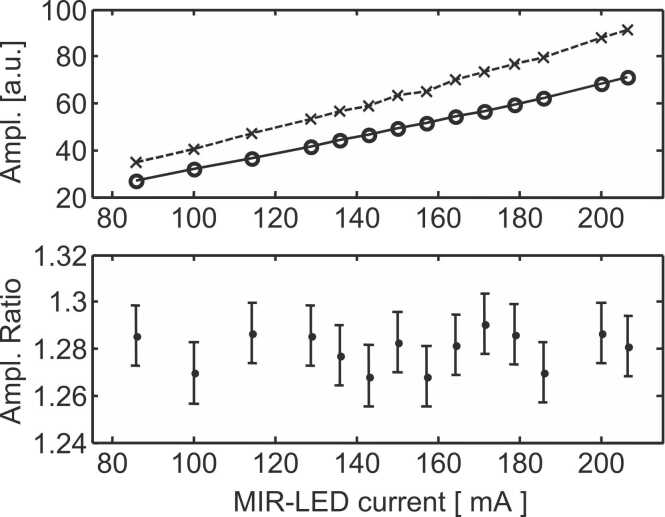
Amplitudes (upper part) and the corresponding ratios (lower part) of the first longitudinal “L1″ (x symbols) and radial “Rad” (o symbols) modes for various MIR-LED driving currents (this current is proportional to the MIR-LED emitted power). The error bars in the lower part correspond to the error (+/- 1 σ) associated with experimentally determined ratios.

Using a ratio instead of an absolute amplitude to determine a concentration has the main advantage of being unaffected by intensity fluctuations of the light source. This is experimentally shown in Fig. 5, where the intensity of the MIR-LED is increased 2.5 times of its initial value without significantly altering the modes amplitude ratio.

**Fig. 6:**
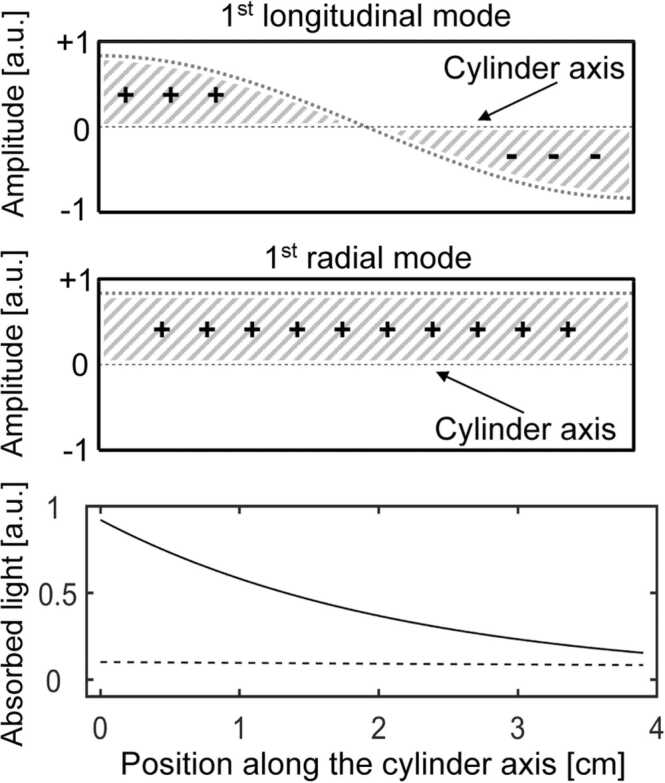
Pressure amplitude of the 1st longitudinal (top) and of the 1st radial (middle) resonant mode for a closed cylindrical pipe. The 1st longitudinal mode exhibits a node in the middle and thus a 180° phase shift while the 1st radial mode has no node and thus a constant phase along the whole cylinder axis. The bottom part shows the amount of absorbed light (in arbitrary units) corresponding to the deposited energy along the 3.9 cm long cylindrical PA cell for an absorption coefficient α of 0.46 cm-1 (corresponding to the peak absorption of 5.6%vv methane diluted in N2 (full line)) while the broken line refers to α = 0.05 cm-1.

The results presented above can be explained by taking into account the location of the deposited energy inside the PA cell which consists of a cylindrical cavity closed at both ends (Fig. 1). The first longitudinal mode of such a cavity has a pressure node in the middle of the cavity and two antinodes, one at each cylinder end (as shown in the top of Fig. 6). The phase shift between the two loops is 180° (i.e. the cylinder barycentre is a symmetry point for the first longitudinal mode) [54]. The acoustic excitation and thus the PA signal depends on the spatial overlap between the absorbed light and the pressure distribution of the acoustic mode of the resonator [4], [54]. Due to the 180° phase shift of the mode respective to the cylinder middle cross section, a light beam equally absorbed within the first and the second half of the cylinder (broken line in the lower part of Fig. 6) will not excite the longitudinal mode. The distribution of the absorbed light along the cylinder is governed by the Lambert-Beer absorption law. For large absorption coefficients (solid line in the lower part of Fig. 6), more light is absorbed within the first half of the cylinder (and the longitudinal mode is excited since the balance due the mode phase shift is not complete. The longitudinal mode amplitude thus strongly and non-linearly increases with the absorption coefficient. Due to this strong signal non-linearity, a strong longitudinal mode signal is measured at a wavelength where the absorption coefficient is large. At wavelengths where the absorption coefficient is small, a negligible signal for the longitudinal mode is obtained even for strong excitation light intensity. Since the first radial acoustic mode has no nodes along the cylinder cavity axis (as shown in the middle part of Fig. 6), the PA signal does not depend on the location of the light absorption, along the cylinder axis. The amplitude of this radial mode thus linearly increases with the amount of light absorbed in the cavity.

As outlined above, the PA signal at a given wavelength (*λ*) for a given mode depends on the spatial overlap between the absorbed light and the spatial amplitude of this mode [4], [54] and is thus proportional to the convolution of the light absorption with the mode amplitude. For the first longitudinal mode (shown in Fig. 6) this leads to a PA signal:

(1) $$\begin{array}{c} S_{PA}\left(\;\alpha_{\lambda }\right)=C_{Cell}\cdot \int_{0}^{L}{\mathrm{Amplitude}}_{\mathrm{Mode}}\left(x\right)\cdot \mathrm{Light}\_{\mathrm{intensity}}_{\mathrm{Absorbed}}\left(x\right)\;dx\\ =C_{Cell}\cdot \int_{0}^{L}\cos\left(\frac{2\pi }{L}x\right)\cdot \left(I_{o,\lambda }\;\alpha_{\lambda }\;e^{-\alpha_{\lambda }x}\right)\;dx\\ \;=C_{Cell}\cdot \frac{I_{0,\lambda }\;{\alpha_{\lambda }}^{2}}{{\alpha_{\lambda }}^{2}+{\left(\pi /L\right)}^{2}}\cdot \left(1+e^{-\alpha_{\lambda }L}\right) \end{array}$$

With *C*_*Cell*_: constant representing the mode acoustic response

*L*: length of the cylindrical PA cell
*I*_*o, λ*_: light intensity at the PA cell entrance at the wavelength *λ*
$\alpha_{\lambda }$: light absorption coefficient at the wavelength *λ*

**Fig. 7:**
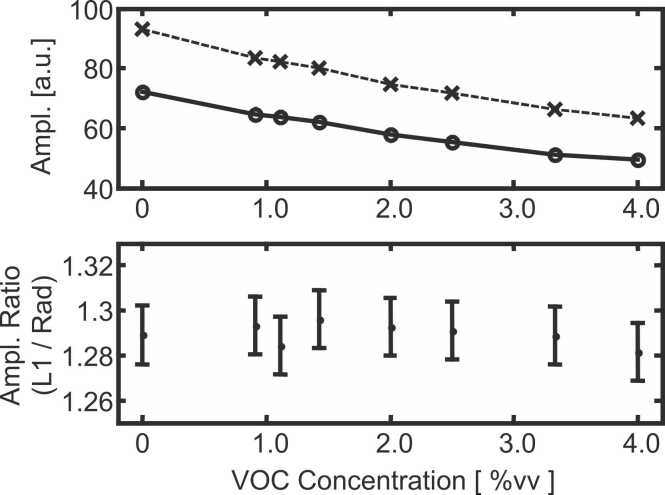
Amplitudes (upper part) and the corresponding ratios (lower part) of the first longitudinal "L1" (x symbols) and radial "Rad" (o symbols) modes for various volatile organic carbon (VOC) concentrations diluted in N2 at atmospheric pressure in the sample cell. The VOC corresponds to a mixture of 86%vv butane (C4H10) and 14%vv propane (C3H8). The error bars in the lower part correspond to the error (+/- 1 σ) associated with experimentally determined ratios.

For the 3.9 cm long PA cell filled with 5.6%vv methane, the integration of *S*_*PA*_( *α*_*λ*_) from Eq. (1) over the full LED emission wavelength range implies that 90% of the PA signal is generated within a small wavelength range around the methane absorption lines corresponding to 2% of the total wavelength region covered by the LED emission. As a comparison, using a typical (e.g. non resonant) NDIR PA setup 90% of the PA signal is generated within a wavelength region corresponding to 12% of the total LED emission range. The PA cell filled with methane acts as a wavelength selective detector similar to a conventional optical correlation setup [50], [52] but with the advantage that the longitudinal mode signal is more selective since its response does not depend on the light intensity at wavelengths at which the absorption coefficient of methane is small. This is exemplified in Fig. 7 which presents the first longitudinal and radial amplitudes for different Volatile Organic Carbons (VOCs, here butane and propane) concentrations present in the sample cell. These two VOCs were chosen since they exhibit broad absorption features and are thus good candidates to test the wavelength selectivity of the proposed sensing scheme. The lower part of Fig. 7 shows that the amplitude ratio longitudinal/radial mode does not significantly depend on the butane/propane concentration in the sample cell when the PA cell is filled with methane diluted in N_2_. The sensing setup is thus highly selective towards methane. A conventional optical correlation-based sensor would have a signal proportional to the absorbed light intensity and thus to the amplitude of the radial mode. As seen in the upper part of Fig. 7, such a sensor presents a 30% signal decrease when the sample cell contains 4%vv of heavier VOCs (more precisely butane (3.4%vv) and propane (0.6%vv)). To reach this 30% signal decrease, 1.6%vv of methane should be introduced in the sample cell in the absence of the other interfering VOCs. This cross-sensitivity (i.e. 4%vv VOC corresponds to 1.6%vv methane) is not observed employing the setup proposed in this work. This is demonstrated in Fig. 7 where no significant ratio change (within the +/- 1% noise) is observed even in the presence of 4%vv VOCs. Based on this fact and since the methane concentration is derived from amplitude ratios (Fig. 4 lower part), the determined methane concentration is not affected by the presence of at least 4%vv VOCs, this means across the whole linear methane concentration range as indicated in the lower part of Fig. 4(i.e. for the range between the LOD and 0.8%vv methane).

## 4 Conclusion

A novel NDIR sensing scheme based on the DME-PAS technique and optical correlation spectroscopy is presented. Since the amplitude ratio of two resonance modes and not the absolute amplitude of the modes is used to derive the concentration of the target gas, the sensor is intrinsically (i.e. without utilizing an external power meter) unaffected by intensity fluctuations of the light source and by any microphone and electronics drifts. Another advantage of the presented technique is its high selectivity, which is even higher than for the case of a conventional optical correlation technique, due to the non-linearity of the longitudinal mode excitation of the PA cell. The first longitudinal and radial modes have been utilized in this work, but other modes and ratios thereof can be used. A MIR-LED light source is used for its robustness, low power consumption and compactness. Furthermore, compared to black-body emitters, the MIR-LED has a high frequency modulation capability without any external moving parts (e.g. mechanical chopper) and requires no MIR bandpass filter to restrict the emission bandwidth. The obtained limit of detection for methane is 25 ppm m^−1^ (1σ, 20 s integration time). This detection limit is obtained with only ca. 40 μW of light intensity entering the PA cell. This light power is quite low for a PA scheme for which usually coherent narrowband sources with power levels of mW to Watt are used [20], [21], [22], [25], [27], [28], [55]. All the above given key advantages of the proposed sensing technique which can also be extended to other target gases clearly demonstrate its high potential for industrial and process gas sensing where cost, selectivity and reliability are of prime importance.

## Declaration of Competing Interest

The authors declare that they have no known competing financial interests or personal relationships that could have appeared to influence the work reported in this paper.

## Data Availability

Data will be made available on request.

## References

[1] Elia A., Lugarà P.M., Di Franco C., Spagnolo V. (2009). Photoacoustic techniques for trace gas sensing based on semiconductor laser sources. Sensors.

[2] Schmid T. (2006). Photoacoustic spectroscopy for process analysis. Anal. Bioanal. Chem..

[3] F.J.M. Harren, S.M. Cristescu, Photoacoustic spectroscopy in trace gas monitoring, in: R.A. Meyers (Ed.), Encyclopedia of Analytical Chemistry, Wiley, 2019.

[4] Miklos A., Hess P., Bozoki Z. (2001). Application of acoustic resonators in photoacoustic trace gas analysis and metrology. Rev. Sci. Instrum..

[5] Sigrist M.W., Chen W., Venables D.S., Sigrist M.W. (2021). Advances in Spectroscopic Monitoring of the Atmosphere.

[6] Palzer S. (2020). Photoacoustic-based gas sensing: a review. sensors.

[7] Hess P. (1983). Resonant photoacoustic spectroscopy. Top. Curr. Chem..

[8] Karbach A., Hess P. (1986). Photoacoustic signal in a cylindrical resonator: theory and laser experiments for CH_4_ and C_2_H_6_. J. Chem. Phys..

[9] Liu L., Huan H., Mandelis A., Zhang L., Guo C., Li W., Zhang X., Yin X., Shao X., Wang D. (2022). Design and structural optimization of T-resonators for highly sensitive photoacoustic trace gas detection. Opt. Laser Technol..

[10] Angeli G.Z., Bozoki Z.J., Miklos A., Lörincz A., Moeckli M., Naegele M., Thöny A., Sigrist M.W. (1991). Design and characterization of a windowless resonant photoacoustic chamber equipped with resonance locking circuitry. Rev. Sci. Instrum..

[11] Suchenek M. (2017). The tracking resonance frequency method for photoacoustic measurements based on the phase response. Int. J. Thermophys..

[12] Bozóki Z., Szabó A., Mohácsi A., Szabó G. (2010). A fully opened photoacoustic resonator based system for fast response gas concentration measurements. Sens. Actuators B.

[13] El-Busaidy S., Baumann B., Wolff M., Duggen L. (2021). Shape optimization of an open photoacoustic resonator. Appl. Sci..

[14] Bernegger S., Sigrist M.W. (1987). Longitudinal resonant spectrophone for CO-laser photoacoustic spectroscopy. Appl. Phys. B.

[15] Gong Z., Gao T., Mei L., Chen K., Chen Y., Zhang B., Peng W., Yu Q. (2021). Ppb-level detection of methane based on an optimized T-type photoacoustic cell and a NIR diode laser. Photoacoustics.

[16] Nägele M., Sigrist M.W. (2000). Mobile laser spectrometer with novel resonant multipass photoacoustic cell for trace-gas sensing. Appl. Phys. B.

[17] Jalink H., Bicanic D. (1989). Concept, design and use of the photoacoustic heat pipe cell. Appl. Phys. Lett..

[18] Harren F.J.M., Bijnen F.G.C., Reuss J., Voesenek L.J., Blom C.M. (1990). Sensitive intracavity photoacoustic measurements with a CO_2_ waveguide laser. Appl. Phys. B.

[19] Liu K., Mei J., Zhang W., Chen W., Gao X. (2017). Multi-resonator photoacoustic spectroscopy. Sens. Actuators B.

[20] Besson J.P., Schilt S., Thévenaz L. (2006). Sub-ppm multi-gas photoacoustic sensor. Spectrochim. Acta A.

[21] Ulasevich A.I., Gorelik A.V., Kouzmouk A.A., Starovoitov V.S. (2013). A miniaturized prototype of resonant banana-shaped photoacoustic cell for gas sensing. Infrared Phys. Technol..

[22] Rouxel J., Coutard J.G., Gidon S., Lartigue O., Nicoletti S., Parvitte B., Vallon R., Zéninari V., Glière A. (2015). Development of a miniaturized differential photoacoustic gas sensor. Proc. Eng..

[23] Bauer R., Stewart G., Johnstone W., Boyd E., Lengden M. (2014). 3D-printed miniature gas cell for photoacoustic spectroscopy of trace gases. Opt. Lett..

[24] Miklos A., Lörincz A. (1989). Windowless resonant acoustic chamber for laser-photoacoustic applications. Appl. Phys. B.

[25] Zeninari V., Kapitanov V.A., Courtois D., Ponomarev Y.N. (1999). Design and characteristics of a differential Helmholtz resonant photacoustic cell for infrared gas detection. Infrared Phys. Technol..

[26] Liu L., Huan H., Li W., Mandelis A., Wang Y., Zhang L., Zhang X., Yin X., Wu Y., Shao X. (2021). Highly sensitive broadband differential infrared photoacoustic spectroscopy with wavelet denoising algorithm for trace gas detection. Photoacoustics.

[27] Mao X., Ji Y., Tan Y., Ye H., Wang X. (2022). High-sensitivity all-optical PA spectrometer based on fast sept laser interferometry. Photoacoustics.

[28] Bonilla-Manrique O.E., Posada Roman J.E., Garcia-Souto J.A., Ruiz-Llata M. (2019). Sub-ppm-level ammonia detection using photoacoustic spectroscopy with an optical microphone based on a phase interferometer. sensors.

[29] Kosterev A.A., Tittel F.K., Serebryakov D.V., Malinovsky A.L., Morozoy I.V. (2005). Applications of quartz tuning forks in spectroscopic gas sensing. Rev. Sci. Instrum..

[30] Patimisco P., Spagnolo V., Meyers R.A. (2021). Encyclopedia of Analytical Chemistry.

[31] Lang Z., Qiao S., Ma Y. (2022). Acoustic microresonator based in-plane quartz-enhanced photoacoustic spectroscopy sensor with a line interaction mode. Opt. Lett..

[32] Duquesnoy M., Aoust G., Melkonian J.M., Levy R., Raybaut M., Godard A. (2021). QEPAS sensor using a radial resonator. Appl. Phys. B.

[33] He Y., Ma Y., Tong Y., Yu X., Tittel F.K. (2019). Ultra-high sensitive light-induced thermoelastic spectroscopy sensor with a high Q-factor quartz tuning fork and a multipass cell. Opt. Lett..

[34] Milde T., Hoppe M., Tatenguem H., Rohling H., Schmidtmann S., Honsberg M., Schade W., Sacher J. (2021). QEPAS sensor in a butterfly package and its application. Appl. Opt..

[35] Lin C., Liao Y., Fang F. (2019). Trace gas detection system based on all-optical quartz-enhanced photoacoustic spectroscopy. Appl. Spectrosc..

[36] Sgobba F., Sampaolo A., Patimisco P., Giglio M., Menduni G., Ranieri A.C., Hoelzl C., Rossmadl H., Brehm C., Mackowiak V., Assante D., Ranieri E., Spagnolo V. (2022). Compact and portable quartz-enhanced photoacoustic spectroscopy sensor for carbon monoxide environmental monitoring in urban areas. Photoacoustics.

[37] Shang Z., Li S., Li B., Wu H., Sampaolo A., Patimisco P., Spagnolo V., Dong L. (2022). Quartz-enhanced photoacoustic NH_3_ sensor exploiting a large-prong-spacing quartz tuning fork and an optical fiber amplifier for biomedical applications. Photoacoustics.

[38] Hu Y., Qiao S., He Y., Lang Z., Ma Y. (2021). Quartz-enhanced photoacoustic-photothermal spectroscopy for trace gas sensing. Opt. Express.

[39] Pan Y., Dong L., Wu H., Ma W., Zhang L., Yin W., Xiao L., Jia S., Tittel F.K. (2019). Cavity-enhanced photoacoustic sensor based on a whispering-gallery-mode diode laser. Atmos. Meas. Tech..

[40] Menduni G., Zifarelli A., Sampaolo A., Patimisco P., Giglio M., Amoroso N., Wu H., Dong L., Bellotti R., Spagnolo V. (2022). High concentration methane and ethane QEPAS detection employing partial least squares regression to filter out energy relaxation dependence on gas matrix composition. Photoacoustics.

[41] Laurila T., Cattaneo H., Koskinen V., Kauppinen J., Hernberg R. (2005). Diode laser-based photoacoustic spectroscopy with interferometrically-enhanced cantilever detection. Opt. Express.

[42] Kuusela T., Kauppinen J. (2007). Photoacoustic gas analysis using interferometric cantilever microphone. Appl. Spectrosc. Rev..

[43] Tomberg T., Hieta T., Vainio M., Halonen L. (2019). Cavity-enhanced cantilever-enhanced photoacoustic spectroscopy. Analyst.

[44] Karhu J., Philip H., Baranov A., Teissier R., Hieta T. (2020). Sub-ppb detection of benzene using cantilever-enhanced photoacoustic spectroscopy with a long-wavelength infrared quantum cascade laser. Opt. Lett..

[45] Fatima M., Hausmaninger T., Tomberg T., Karhu J., Vainio M., Hieta T., Genoud G. (2021). Radiocarbon dioxide detection using cantilever-enhanced photoacoustic spectroscopy. Opt. Lett..

[46] Rey J.M., Sigrist M.W. (2007). Differential mode excitation photoacoustic spectroscopy: a new photoacoustic detection scheme. Rev. Sci. Instrum..

[47] Rey J.M., Sigrist M.W. (2008). New differential mode excitation photoacoustic scheme for near-infrared water vapour sensing. Sens. Actuators B.

[48] Rey J.M., Sigrist M.W. (2008). Simultaneous dual-frequency excitation of a resonant photoacoustic cell. Infrared Phys. Technol..

[49] Rey J.M., Romer C., Gianella M., Sigrist M.W. (2010). Near-infrared resonant photoacoustic gas measurement using simultaneous dual-frequency excitation. Appl. Phys. B.

[50] Luft K.F. (1943). Ueber eine neue Methode der registrierenden Gasanalyse mit Hilfe der absorption ultraroter Strahlen ohne spektrale Zerlegung. Z. Tech. Phys..

[51] Fastie W.G., Pfund A.H. (1947). Selective infra-red gas analyzer. J. Opt. Soc. Am..

[52] Chan S.H. (1996). Measurement of concentrations of transient gases using a conventional NDIR analyser. Meas. Sci. Technol..

[53] Johnson T.J., Sams R.L., Sharpe S.W. (2004). The PNNL quantitative infrared database for gas-phase sensing: a spectral library for environmental, hazmat, and public safety standoff detection. Proc. SPIE.

[54] Morse P.M., Ingard K.U. (1986).

[55] Wolff, Rhein S., Bruhns H., Nähle L., Fischer M., Koeth J. (2013). Photoacoustic methane detection using a novel DFB-type diode laser at 3.3 μm. Sens. Actuators B.

